# Effects of the Active Kids voucher program on children and adolescents’ physical activity: a natural experiment evaluating a state-wide intervention

**DOI:** 10.1186/s12889-020-10060-5

**Published:** 2021-01-11

**Authors:** Bridget C. Foley, Katherine B. Owen, Adrian E. Bauman, William Bellew, Lindsey J. Reece

**Affiliations:** SPRINTER (Sport and Active Recreation Intervention & Epidemiology Research Group), Prevention Research Collaboration, Sydney School of Public Health, Faculty of Medicine and Health, D17 Charles Perkins Centre, The University of Sydney, Level 6, the Hub, Camperdown, NSW 2006 Australia

**Keywords:** Children, Adolescents, Financial incentive, Voucher, Organized sport, Physical activity, Leisure-time, Evaluation, Policy, Behavior change

## Abstract

**Background:**

There is an urgent need for scaled-up effective interventions which overcome barriers to health-enhancing physical activity for children and adolescents. In New South Wales (NSW), Australia, the state government implemented a universal voucher program, ‘Active Kids’ to support the cost of structured physical activity registration for school-enrolled children aged 4.5–18 years old. The objective of this study was to understand the effects a financial incentive intervention delivered in a real-world setting has on children and adolescent’s physical activity participation.

**Method:**

In 2018, all children and adolescents registered for an Active Kids voucher provided sociodemographic characteristics, physical activity and research consent. This prospective cohort study used an online survey with validated items to measure physical activity and other personal and social factors in children and adolescents who used an Active Kids voucher. Generalized linear mixed models were used to examine changes from registration to after voucher use at ≤8 weeks, 9–26 weeks and ≥ 6 months.

**Results:**

Study participants reported increasing their days achieving physical activity guidelines from 4.0 days per week (95%CI 3.8, 4.2) at registration (*n* = 37,626 children) to 4.9 days per week (95%CI 4.7, 5.1) after 6 months (*n* = 14,118 children). Increased physical activity was observed for all sociodemographic population groups. The voucher-specific activity contributed 42.4% (95%CI 39.3, 45.5) to the total time children participated in structured physical activities outside of school. Children and adolescents who increased to, or maintained, high levels of activity were socially supported to be active, had active parent/caregivers, had better concentration and were overall happier than their low-active counterparts.

**Conclusion:**

The Active Kids program significantly increased children’s physical activity levels and these increases continued over a six-month period. The Active Kids voucher program shows promise as a scaled-up intervention to increase children and adolescents’ physical activity participation.

**Trial registration:**

Australian New Zealand Clinical Trial Registry ACTRN12618000897268, approved May 29th, 2018 - Retrospectively registered.

**Supplementary Information:**

The online version contains supplementary material available at 10.1186/s12889-020-10060-5.

## Background

Increasing the amount of physical activity children and adolescents achieve each day is a global priority [1]. It is recommended that all children aged 5–17 years accumulate at least 60 min of moderate to vigorous physical activity each day [2, 3]. Adhering to these guidelines is associated with enhanced cardiorespiratory and musculoskeletal health and fitness, improved body composition, academic achievement and cognition, quality of life, mental health, social and emotional behaviours in children [2, 4]. At the societal level, increased physical activity produces co-benefits across many Sustainable Development Goals, as noted by the World Health Organisation, including reduction of premature mortality from non-communicable disease prevention, contribution to job creation for young people and reduction in social inequalities through promoting fairness and inclusion [1, 5]. Worldwide, more than 80% of adolescents (11–17 years old) are not meeting physical activity recommendations, while Australian adolescents are among the least active with just 11% achieving recommended levels of physical activity for health [2, 6]. There is an urgent need to implement scaled-up effective interventions to improve children and adolescent’s health-enhancing physical activity behaviours.

Participation in structured physical activity is one-way to increase achievement of the recommended physical activity guidelines [7, 8]. Structured physical activities include opportunities delivered though an organisation, which involve physical exertion, skill and/or hand-eye coordination as the primary focus of the activity [1]; but elements of competition are not essential. These may be undertaken as team or individual pursuits such as sport participation (e.g. Football, Swimming, Athletics, Tennis) and/or active recreation (e.g. Dance, Martial Arts, bush survival skills etc). Participation in structured physical activity programs throughout childhood and adolescence is influenced by multiple barriers and facilitators across individual, interpersonal, community and societal levels [9–11]. Modifiable barriers limiting children and adolescent’s participation in structured physical activity include the cost of registration, equipment and uniforms; access to appropriate and safe opportunities; lack of time and having friends involved [12, 13]. Knowing this, real-world interventions which aim to overcome barriers to structured physical activity participation are urgently required [1].

Financial incentive policies and programs that aim to reduce the cost barrier for children and adolescents may increase participation and retention in structured physical activity [14]. There has been a marked increase in public sector investment for financial incentive programs that directly reduce the cost barrier to structured physical activity participation [15, 16]. To date, heterogenous interventions tested in randomised controlled trials suggest that financial incentives hold promise to get more children active [17–20]. The ACTIVE trial adopted a co-design approach with teenagers, providing them free choice of unstructured activities the vouchers could be used for, which had a positive impact on cardiovascular fitness, cardiovascular health, and perspectives of activity [17, 18]; Dunton tested after-school physical activity programs for primary school children in low-income families which has limited effectiveness [20]; Financial incentives have also been used to promote physical activity in overweight/obese American Indian youth (11–20 years old) resulting in longer session duration but minimal effect on the number of sessions youth participated in [19]. A cross-sectional study of the Government of Canada’s Canadian Fitness Tax Credit, which provided a non-refundable tax credit for structured physical activity programs (including sport and dance) for all children up to 16 years old found the tax credit benefited the wealthier families most [21]. In Australia, it is estimated that families spend AUD $447 annually on structured physical activity, per child [15]. Sport voucher programs have also been implemented by governments in different Australian jurisdictions, each adopting a unique approach, with limited process or outcome evaluation on the effectiveness of this type of intervention [15]. Pragmatic evaluations of large-scale interventions should be undertaken to inform policy and practice [22, 23].

In 2018, the NSW Government allocated $207 million across four years for a universal voucher program, entitled Active Kids [24]. More than 1.2 million school-enrolled children aged between 4.5 and 18 years old were potentially eligible to register for one AUD $100 voucher per calendar year. The voucher aimed to increase structured physical activity participation outside of school by reducing the cost barrier. A complex yet pragmatic quasi-experimental, mixed-methods evaluation was integrated into the design of Active Kids and involves a series of studies [25]. We have previously reported the population awareness and reach of the Active Kids program [26, 27]. The objective of this study was to fill the gap in understanding of the impact of a universal, state-wide financial incentive intervention (Active Kids voucher) on children’s physical activity participation, and the contribution of the voucher to support structured physical activity participation. Personal and social associations with being active were explored to understand whether the voucher influenced underlying contextual factors.

## Method

### Study design

This study is a natural experiment using a prospective cohort study design, nested within the Active Kids state-wide program evaluation [25]. Natural experiments are appropriate when exposure to the intervention of interest has not been manipulated by the researcher and events that occur during the experiment are outside the control of the researcher [23, 28]. Using data collected during the first year of the Active Kids program, we aimed to address the following research questions:

1. Does using an Active Kids voucher increase the number of days per week children participate in physical activity for at least 60 min, and are any increases maintained six months after using the voucher?
2. What proportion of the child’s reported weekly time and annual expenditure on structured physical activities does the Active Kids voucher contribute towards?
3. Are changes in physical activity participation after voucher use associated with personal and social factors in children’s lives?

### Active Kids program description

The Active Kids program is a state-wide, whole-of-government initiative led by the NSW Government Office of Sport [29]. It provides all school-enrolled children aged between 4.5 and 18 years old access to a financial voucher (valued up to AUD $100) to reduce the cost of registration or membership in an approved structured program of at least 8 weeks’ duration which involves moderate or vigorous levels of physical activity. Eligible voucher programs include team sports, individual sports, swimming lessons, structured fitness programs, active recreation programs and dance, which were not held during school time or delivered by schools.

The Active Kids program is administered through a bespoke government platform. Upon registration, each child receives a unique voucher code which can be redeemed with an Active Kids provider to reduce the cost of registration or membership. Activity providers must also register with the NSW Government Office of Sport for Active Kids accreditation to enable them to redeem and Active Kids voucher. Once the accredited provider redeems the voucher through this platform, the child’s voucher status changes from available to redeemed within the platform. Further programmatic details of Active Kids can be accessed here: https://www.sport.nsw.gov.au/sectordevelopment/activekids

### Inclusion criteria

**Fig. 1:**
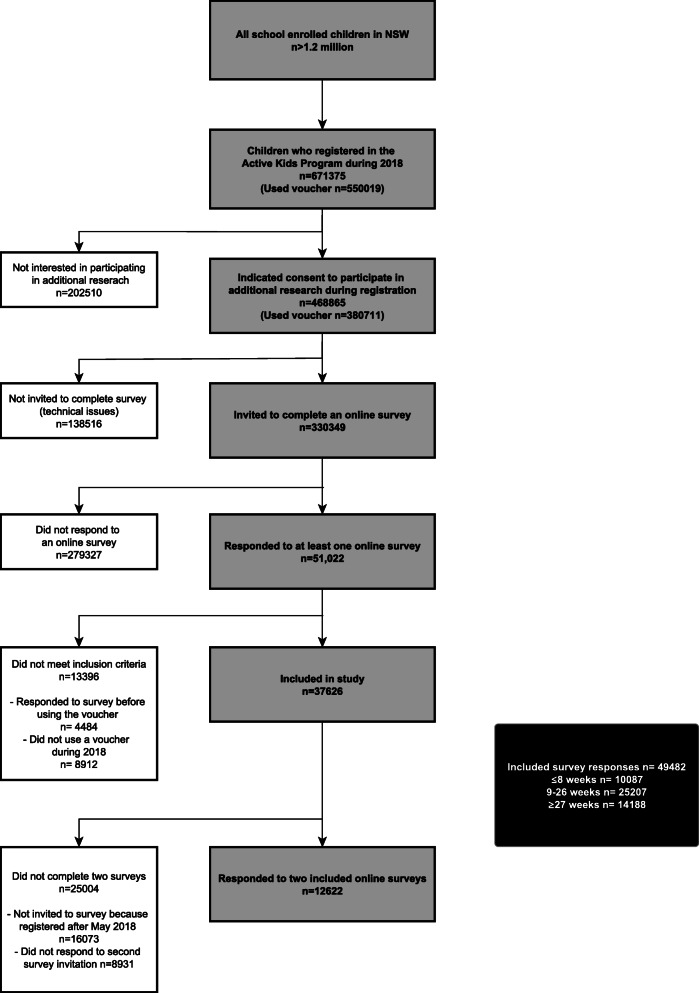
Participant recruitment flow

All children registered in the Active Kids program who provided written active consent (often by-proxy through parent/guardian) during the online Active Kids registration were eligible to be included in the study. Consent was indicated through selection of a tick box within the online Active Kids registration on the bespoke government webpage. Data regarding children were included if a response to the online survey was received after the child’s Active Kids voucher had been redeemed. Participant flow for this study is shown in Fig. 1.

### Measurement

The research protocol outlined that everyone who agreed to participate in research during the voucher registration process would be sent an invitation to an online survey eight and eighteen weeks after they had redeemed the Active Kids voucher using a rolling recruitment method. A decision was made, due to technical issues connecting the database and survey platform, to adjust the protocol and invite all participants (often by-proxy through parent/guardian) to complete a survey at two time points to assess their physical activity participation (May 2018 and November 2018) (see Fig. 1). The STROBE checklist and the Checklist for Reporting Results of Internet E-Surveys were used to ensure quality reporting of our methods [30, 31].

Invitations to the online survey were emailed from a NSW government domain on behalf of the researchers. After the participant information statement was read and understood, participants (often by-proxy through parent/guardian) indicated consent through completion of the online survey. The survey was hosted on Form Assembly in May and by Australian Survey Research platform (Survey Manager) in November. Surveys remained open to those invited for 3 weeks with a reminder sent to those who had not completed the survey after 2 weeks. Partially completed surveys were included in the analysis with missing data excluded from analysis. No incentives or rewards were offered to people who participated in the survey.

### Instruments

#### Registration platform

Provision of sociodemographic information and primary outcome (physical activity) data were mandatory during registration for an Active Kids voucher. Sociodemographic data fields in the registration platform included the child’s name, date of birth, sex, Indigenous status, disability status, language spoken at home, postcode. Date of birth was used to categorize children into four age groups (4–8 years; 9–11 years; 12–14 years; 15–18 years) which are consistent with the developmental stages for children and adolescents, defined by the Sport sector in Australia [32]. Socio-economic status of children was derived from their reported postcode using the Australian Bureau of Statistic’s Socio-Economic Index For Areas Index of Relative Disadvantage [33]. National percentiles were then categorized into quartiles. Geographic location was classified using the reported postcode and determined using Accessibility/Remoteness Index of Australia Plus [34]; Outer regional and remote were combined in the analysis. Height and weight fields were included in the registration platform however the fields were not mandatory during 2018. Body Mass Index z-scores (BMI) were calculated using the height and weight of the child reported during the registration process. Children were classified as thin, healthy weight, overweight or obese using the International Obesity Task Force cut points [35].

The primary outcome for this study was the number of days the child participated in at least 60 min of physical activity. This was assessed using a proxy self-report single-item 7-day recall validated question [36, 37]:

“In a typical week, how many days was the child physically active for at least 60 minutes? *This could be made up of different activities including walking, cycling to school, and sport at lunchtime or an exercise class.*”

Annual sport participation was collected in the registration form using an AusPlay survey item [38].

#### Online survey

The online survey was designed to be completed by the child’s parent/caregiver, with the child present or by a child able to provide informed consent (16–18 years old). To minimise bias, it was recommended the child was present and specific instructions were to ensure the child was asked for their response. The survey was developed specifically for the evaluation of the Active Kids program using validated self-report or proxy-report items for measurement where possible [39]. See Additional File 1 for the survey items included in the May 2018 and November 2018 surveys.

The 7-day recall of the child’s physical activity used in the registration platform was repeated within the survey [36, 37]. Time in the past 7 days spent participating in structured physical activity, and in the activity where the child used the Active Kids voucher, were collected using modified items from the National sport surveillance survey AusPlay; these included days per week, sessions per week and duration of each session, as well as the AusPlay item for annual cost of sport participation [38].

Personal and social factors which may moderate the effect of the voucher were also measured through the online survey. Children’s self-efficacy and enjoyment of being physically active [40, 41], ease of locating places to be physically active [42] and social influences on child’s physical activity [43] were measured using validated items. Adults were asked to identify the recommended minutes of physical activity children should accumulate each day [44], their own physical activity participation [45] and their own organized sport participation in the previous seven days [38] using validated survey items.

### Data analysis

All analyses were conducted using IBM SPSS Statistics for Windows, Version 25 (IBM Corp., Armonk, N.Y., USA). The descriptive characteristics of all children registered for a voucher, along with subsamples who used an Active Kids voucher, and the cohort study participants were analysed. Due to the large sample size, significant differences were observed between all groups, therefore, proportional reporting ratios (PRR) were calculated to determine the magnitude of differences between all children registered in the program and study participants.

#### Timepoints

After registering for a voucher, the participant and their context determined where and when they redeemed the voucher. All participants (often by-proxy through parent/guardian) provided primary outcome data at registration, and at least once after using their voucher through responding to the online survey. Each participant’s voucher redemption date was recorded as the date the provider redeemed the voucher in the bespoke government administration platform. Three post-voucher categories were generated using the difference in weeks between the voucher redemption date and the median date in the data collection period for each survey. Voucher activities were required to last at least eight weeks; therefore, the first category was defined by surveys completed within ‘8 weeks or less’ after voucher redemption. An interim time point of 9–26 weeks was generated and the final timepoint was responses ≥27 weeks (≥6 months) after voucher redemption. This categorisation enabled within person analysis to be undertaken for the primary outcome.

#### Physical activity outcomes

Bivariate generalized linear mixed models were used to assess the associations between sociodemographic characteristics and the number of days the child participated in at least 60 min of physical activity at registration. A multivariable generalized linear mixed model was used to examine changes in the number of days the child participated in physical activity for at least 60 min over time (from registration to ≤8 weeks, 9–26 weeks and 6 months+ after voucher use), adjusting for all sociodemographic characteristics. Sociodemographic characteristics included sex, age, Indigenous status, disability status, language spoken at home, socio-economic status, geographic location and BMI. Interactions between physical activity and sociodemographic characteristics over time were also tested. For the interaction results, the Bonferroni correction for multiple comparisons was applied.

A multivariable generalized linear mixed model was used to determine what contribution using the Active kids voucher had on children’s sport participation and annual expenditure. This model adjusted for all sociodemographic characteristics and the Bonferroni correction for multiple comparisons was applied.

#### Personal and social associations with increased activity after voucher use

Generalized linear mixed models were conducted to assess associations between increased physical activity and personal and social influences on the child. We categorised children who increased physical activity in two different ways to do this. Model 1 used observations from children who were active on ≥5 days per week at any survey time point, with the reference group being children who were active < 5 days per week, in order to strengthen our understanding of high activity levels and associated personal and social contextual factors. The ≥5 day cut-point was selected rather than a 7-day cut-point to compare the most active to the least active children. Model 2 compared those who increased the number of days they achieved 60 min of physical activity from the number of days reported at registration, against those who maintained the same number of days (0–7 days) or decreased days achieving guidelines. Children who achieved the physical activity guideline and maintained this over time were considered in the reference group to strengthen our understanding of those who increased their physical activity after using an Active Kids voucher in Model 2.

## Results

During the first year of implementation of the Active Kids program (2018) in NSW, 671,375 children registered for an Active Kids voucher. Of these, 550,019 children (81.9%) used an Active Kids voucher, from whom 380,711 (69.2%) indicated consent (by-proxy) to participate in research (Fig. 1). Table 1 presents the demographic characteristics of children registered in the Active Kids program and those included in this cohort study (*n* = 37,626). Consent through survey participation was indicated by-proxy for most participants, with less than 1% of children aged over 16 completed their own survey.

**Table 1:** Participant sociodemographic characteristics at registration compared to the research cohort

	All children ***N***%	Children who used a voucher ***N***%	Used a voucher / All	Participants who completed one survey N%	Survey one / All	Participants who completed two surveys N%	Survey two / All
	***N = 671,375*** **(100%)**	***N = 550,019 (81.9%)***	***PRR (95%CI)***	***N =*** 37,626	***PRR (95%CI)***	***N =*** 12,622	***PRR (95%CI)***
Sex*							
Male	**361,852 (54.0)**	300,103 (54.6)	1.01 (1.01–1.02)	19,607 (52.2)	0.97 (0.96, 0.98)	7140(56.6)	1.05 (1.03, 1.06)
Female	**308,543 (46.0)**	249,133 (45.4)	0.99 (0.98–0.99)	17,973 (47.8)	1.04 (1.03, 1.05)	5472(43.4)	0.94 (0.93, 0.96)
**Age**							
4–8 years	**269,457 (40.1)**	226,386 (41.2)	1.03 (1.02–1.03)	16,388 (43.6)	1.09 (1.08, 1.09)	5005(39.7)	0.99 (0.97, 1.00)
9–11 years	**185,931 (27.7)**	156,364 (28.4)	1.03 (1.02–1.03)	9945 (26.4)	0.95 (0.94, 0.97)	3476(27.5)	0.99 (0.98, 1.01)
12–14 years	**138,063 (20.6)**	110,621 (20.1)	0.98 (0.97–0.98)	7376(19.6)	0.95 (0.94, 0.97)	2742(21.7)	1.06 (1.04, 1.08)
15–18 years	**77,924 (11.6)**	56,648 (10.3)	0.89 (0.88–0.89)	3917(10.4)	0.90 (0.88, 0.91)	1399(11.1)	0.95 (0.93, 0.98)
**Aboriginal/Torres Strait Islander**							
No	**626,688 (93.3)**	514,483(93.5)	1.00 (1.00–1.00)	35,644(94.7)	1.01 (1.01, 1.02)	11,961(94.8)	1.02 (1.01, 1.03)
Yes	**36,129 (5.4)**	28,618 (5.2)	0.97 (0.96–0.97)	1533(4.1)	0.76 (0.73, 0.78)	512 (4.1)	0.76 (0.72, 0.80)
Prefer not to say	**8558 (1.3)**	6918(1.3)	0.99 (0.97–1.00)	449(1.2)	0.94 (0.88, 0.91)	149(1.2)	0.92 (0.84, 1.00)
**Primary language spoken at home**							
English	**621,235 (92.5)**	513,793(93.4)	1.01 (1.01–1.01)	34,651 (92.1)	1.00 (0.99, 1.00)	11,999(95.1)	1.03 (1.02, 1.04)
Language other than English	**50,140 (7.5)**	36,226(6.6)	0.88(0.87–0.89)	2975(7.9)	1.06 (0.98, 1.08)	623(4.9)	0.65 (0.61, 0.69)
**Identified disability**							
No	**644,658 (96.1)**	530,202(96.5)	1.00(1.00–1.01)	36,085(96.0)	1.00 (0.99, 1.01)	12,177(96.7)	1.01 (1.00, 1.02)
Yes	**17,715 (2.6)**	12,772 (2.3)	0.88(0.87–0.89)	1077(2.9)	1.08 (0.97, 1.12)	305(2.4)	0.92 (0.86, 0.98)
Prefer not to say	**8277 (1.2)**	6420(1.2)	0.95(0.93–0.96)	426(1.1)	0.92 (0.95, 0.97)	113(0.9)	0.75 (0.66, 0.84)
**Socio-economic status^**							
1st Quartile (Most Disadvantaged	**99,583 (16.6)**	76,900(15.7)	0.94 (0.94–0.95)	4523 (13.2)	0.81 (0.83, 0.98)	1257(11.6)	0.70 (0.67, 0.73)
2nd Quartile	**140,302(23.4)**	116,191(23.7)	1.01(0.01–0.02)	7979(23.3)	1.01 (1.03, 0.99)	2617(24.2)	1.03 (1.01, 1.05)
3rd Quartile	**158,783 (26.5)**	130,315(26.5)	1.00(1.00–1.01)	9502(27.7)	1.07 (1.08, 0.99)	2925(27.0)	1.02 (1.00, 1.04)
4th Quartile (Least Disadvantaged)	**200,566 (33.5)**	167,753(34.2)	1.02(1.02–1.02)	12,289(35.8)	1.09 (1.10, 0.99)	4016(37.1)	1.11 (1.09, 1.13)
**Geographic location^**							
Major city	**440,793 (73.5)**	359,235 (73.1)	0.99 (0.99–1.00)	25,593(74.5)	1.04 (0.99, 1.04)	7898(72.9)	0.99 (0.98, 1.00)
Inner regional	**126,594 (21.1)**	105,485(21.5)	1.02(1.01–1.02)	7062(20.6)	1.00 (0.99, 1.01)	2401(22.2)	1.05 (1.03, 1.07)
Outer regional and remote	**32,622 (5.4)**	27,035(5.5)	1.01(1.00–1.02)	1681(4.9)	0.92 (0.97, 0.94)	530(4.9)	0.91 (0.87, 0.95)
Body Mass Index (BMI) classification, reported at baseline **							
Thin	**35,357 (11.5)**	29,815(11.6)	1.03 (1.02–1.04)	2557(12.3)	1.29 (0.98, 1.31)	971(11.8)	1.03 (1.00, 1.06)
Healthy weight	**195,166 (63.7)**	165,065(64.1)	1.03(1.03–1.04)	13,490(64.8)	1.23 (0.99, 1.24)	5427(65.9)	1.03 (1.01, 1.05)
Overweight	**52,675 (17.2)**	43,724(17.0)	1.01(1.01–1.02)	3395(16.3)	1.15 (0.98, 1.17)	1342(16.3)	0.95 (0.92, 0.98)
Obese	**23,252(7.6)**	18,786(7.3)	0.99(1.98–1.00)	1383(6.6)	1.06 (0.97, 1.09)	491(6.0)	0.79 (0.74, 0.84)
**Physical activity, reported at baseline#**							
Insufficiently active	**524,334(80.2)**	427,349 (79.6)	0.99(0.99–1.00)	29,119(78.7)	0.99 (0.99, 1.00)	9505(76.2)	0.96 (0.95, 0.98)
Met physical activity guidelines	**129,228 (19.8)**	109,710(20.4)	1.04(1.03–1.04)	7859(21.3)	1.09 (0.99, 1.10)	2965(23.8)	1.20 (1.18, 1.22)
**Childs annual organised sport and physical activity participation, reported at baseline#**							
Non-participant	**12,238 (1.9)**	7193 (1.4)	0.72(0.70–0.73)	594 (1.6)	0.87 (0.95, 0.91)	23(0.2)	0.11 (−0.09, 0.31)
Casual participant (<less than once per week)	**151,675 (24.0)**	114,237(22.1)	0.92(0.92–0.92)	7348(20.4)	0.86(0.99, 0.88)	1988(16.4)	0.68 (0.66, 0.70)
Regular participant (1–1.9 sessions per week)	**217,878 (34.5)**	182,579(35.2)	1.02(1.02–1.03)	12,730(35.3)	1.04 (0.99, 1.05)	4237(34.9)	1.01 (0.99, 1.03)
Regular participant (2–3.9 session per week)	**147,616 (23.4)**	126,284(24.4)	1.04(1.04–1.05)	9012(25.0)	1.09 (0.99, 1.10)	3328(27.4)	1.17 (1.15, 1.19)
Committed participant (> 4 session per week)	**101,290 (16.1)**	87,677(16.9)	1.06(1.05–1.06)	6354(17.6)	1.12 (0.99, 1.13)	2567(21.1)	1.31 (1.29, 1.33)

*Participants did not report sex of the child at birth (*n* = 980, < 0.2%) ^Some postcodes were missing or invalid (*n* = 72,141, 11% for socioeconomic status) (*n* = 71,366, 11% for geographic location); **Reporting height and weight was provided voluntarily (*n* = 364,925, 54% missing); #Participant reported being ‘unsure’ at registration(n = 17,813, 2.7% for physical activity) (n = 40,678, 6% for annual organised sport and physical activity)

Study participants were similar to all children who registered for a voucher (Table 1). Proportional reporting ratios showed children who responded to at least one survey were less likely to be older, identified as Aboriginal/Torres Strait Islander, lived in the most disadvantaged areas, lived in outer regional and remote areas, were obese and participated in sport less than once a week. Children who responded to two surveys were less likely to speak a primary language other than English at home or lived in the most disadvantaged socio-economic quartile. Study participants were slightly more physically active than all children who registered in the Active Kids program, with 21.3% of study participants meeting physical activity guidelines compared to 19.8% of all children (Table 1); unadjusted mean days achieving physical activity guidelines at registration were 4.5 days (SD 1.8) compared to 4.4 days (SD 1.8) respectively.

At registration, all sociodemographic correlates, except Indigenous status, were significantly associated with physical activity days in the last week. Significantly lower physical activity levels were observed for children who were female, older (12+ years), spoke a language other than English at home, identified as having a disability, lived in socio-economically disadvantaged areas, lived in major cities or were above a healthy weight, compared to their counterparts before engaging in the Active Kids program (Table 2).

**Table 2:** Bivariate analysis of mean days of 60-min physical activity at registration in the cohort (*n* = 37,375)

		Mean days (95% Confidence Interval)	Contrast	Significance
All		4.46 (4.44, 4.48)	–	–
Sex	Male	4.65 (4.62, 4.67)	Ref	Ref
	Female	4.26 (4.23, 4.28)	−0.39	< 0.0001
Age	4–8 years	4.50 (4.48, 4.53)	Ref	Ref
	9–11 years	4.50 (4.47, 4.54)	0.00	0.875
	12–14 years	4.39 (4.35, 4.43)	−0.12	< 0.0001
	15–18 years	4.29 (4.23, 4.35)	−0.22	< 0.0001
Aboriginal/Torres Strat Islander	No	4.46 (4.43, 4.47)	Ref	Ref
	Yes	4.55 (4.45, 4.64)	0.09	0.061
Language spoken at home	English	4.51 (4.49, 4.53)	Ref	Ref
	Other	3.85 (3.78, 3.92)	−0.66	< 0.0001
Disability	No disability	4.48 (4.46, 4.50)	Ref	Ref
	Disability	3.97 (3.86, 4.09)	−0.50	< 0.0001
Socio-economic status	1st Quartile Most disadvantaged	4.24 (4.19, 4.30)	Ref	Ref
	2nd Quartile	4.53 (4.49, 4.57)	0.29	< 0.0001
	3rd Quartile	4.40 (4.37, 4.44)	0.16	< 0.0001
	4th Quartile Least Disadvantaged	4.52 (4.49, 4.56)	0.28	< 0.0001
Geographic location	Major City	4.40 (4.37, 4.42)	Ref	Ref
	Regional	4.61 (4.57, 4.65)	0.21	< 0.0001
	Outer regional and remote	4.69 (4.60, 4.77)	0.29	< 0.0001
Body Mass index	Thin	4.48 (4.57, 4.71)	−0.01	0.799
	Healthy weight	4.65 (4.62, 4.68)	Ref	Ref
	Overweight	4.40 (4.34, 4.46)	−0.25	< 0.0001
	Obese	4.13 (4.03, 4.22)	−0.52	< 0.0001

* Some participants reported being unsure of their child’s physical activity at registration

### Influence of the Active Kids voucher on achievement of recommended physical activity guidelines

**Fig. 2:**
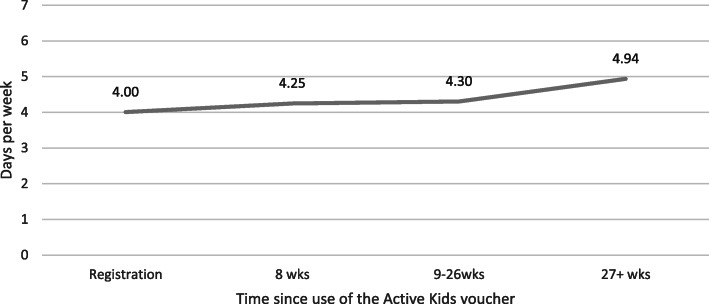
Changes in mean days doing 60 min of physical activity (*N* = 37,375, with 79,038 observations)

Participation in the Active Kids program increased the mean days children participated in at least 60 min of physical activity from 4.00 days (95% CI 3.80, 4.21) at registration to 4.94 days (95%CI 4.73, 5.15) after 6 months (*P* < 0.0001). Within eight weeks, there was a 0.25 mean increase (P < 0.0001) in the number of days the child participated in 60 min of physical activity, and a 0.30 day increase from registration to after 9–26 weeks (Fig. 2). The multivariable coefficient results are provided in Additional File 2.

**Fig. 3:**
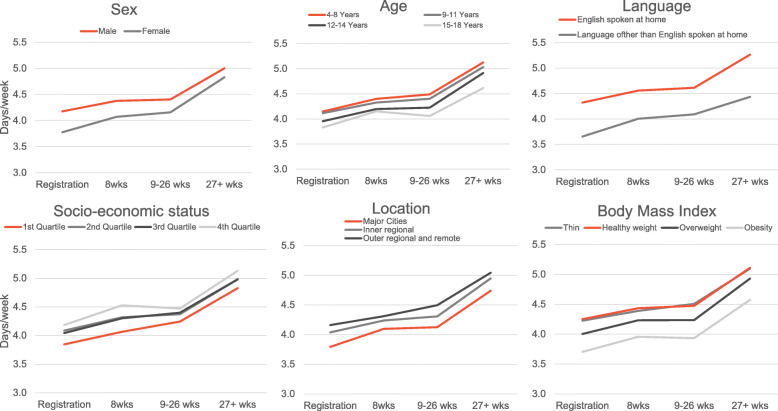
Interactions between days achieving 60 min of physical activity and significant sociodemographic correlates

Significant interactions with time were found for children by sex (F = 16.647, *P* < 0.0001), age (F = 9.316, *P* < 0.0001), language spoken at home (F = 9.316, *P* < 0.0001), socio-economic status (F = 6.879, *P* < 0.0001), location (F = 8.123, *P* < 0.0001) and BMI (F = 7.013, *P* < 0.0001) (Fig. 3). There were no significant interactions with time for children by disability status (F 1.404, *P* = 0.155). The disparity in days achieving physical activity guidelines between females and males at registration (0.4 days) reduced after 6 months to 0.2 days, with more significant impacts among female participants. Disparities increased for older children (15–18-year old) compared to younger children from 0.3 days at registration to 0.5 days after six months. Similarly, children who spoke a language other than English at home were 0.6 days less active at registration, and this increased to 0.8 days less active than their English-speaking counterparts. Differences between the most and least disadvantaged groups were greatest in the ≤8-week period (0.4 days), with the least disadvantaged group increasing more, however disparities returned previous levels after 6 months. Children living in the city and obese children, responded positively when the voucher was in use (≤8-week period) then disparities returned to previous levels over time (Fig. 3).

### Contribution of the voucher to weekly time and annual expenditure on structured physical activities

The top 10 structured physical activities study participants used their voucher for were Football (Soccer) (28.4%), Netball (10.6%), Swimming (10.2%), Multisport (7.9%), Dance (7.3%), Rugby league (6.0%), Gymnastics (3.7%), Basketball (3.6%), Australian Football League (AFL) (3.1%) and Rugby Union (2.9%). After using an Active Kids voucher, the mean weekly duration children participated in structured physical activity outside of school was 5.97 h (SD 6.62), with a mean contribution of 2.40 h (SD 3.28) to the total from the voucher activity. The selected Active Kids voucher activity contributed 42.37% [95%CI 39.28, 45.49] of the total time children reported participating in structured physical activities (Table 3). The voucher made a greater contribution to participation for Active Kids who were 15–18 years old, Aboriginal/Torres Strait Islander, spoke a language other than English, had a disability, lived in socio-economically disadvantaged areas, or were obese (not overweight) (Table 3).

**Table 3:** Contribution of the Active Kids voucher to weekly time doing structured physical activity

		Hours per week doing structured physical activity (voucher and non-voucher activity) *n* = 35,297 x̄ Hours (95%CI)	Voucher activity contribution to total weekly minutes *n* = 27,737 x̄ Contribution (95%CI)
Sex	Male	6.33 (5.71, 6.95)	43.80% (41.64, 45.97)
	Female	6.43 (5.81, 7.06)	43.15% (40.98, 45.33)
Age group	4–8 years	3.39 (2.51, 4.28)	42.65% (39.59, 45.69)
	9–11 years	5.48 (4.59, 6.37)	42.51% (39.45, 45.57)
	12–14 years	7.29 (6.40, 8.18)	43.37% (40.31, 46.44)
	15–18 years	7.90 (6.99, 8.81)	44.93% (41.82, 48.03)
Aboriginal/Torres Strait Islander	No	5.42 (4.55, 6.30)	42.85% (39.85, 45.85)
	Yes	6.03 (5.11, 6.96)	45.53% (42.35, 48.71)
Primary language spoken at home	English	6.52 (5.64, 7.39)	42.47% (39.47, 45.49)
	Language other than English	5.51 (4.60, 6.42)	44.24% (41.10, 47.39)
Identified disability	No	6.55 (5.82, 7.28)	42.02% (39.47, 44.56)
	Yes	5.75 (4.93, 6.57)	43.44% (40.59, 46.32)
Socio-economic status	1st Quartile *Most Disadvantaged*	6.55 (5.54, 7.57)	45.63% (42.11, 49.16)
	2nd Quartile	6.15 (5.14, 7.16)	44.62% (41.10, 48.11)
	3rd Quartile	6.16 (5.15, 7.17)	44.20% (40.69, 47.69)
	4th Quartile *Least Disadvantaged*	5.95 (4.94, 6.96)	40.88% (37.35, 44.40)
Location	Major city	6.12 (5.18, 7.07)	42.73% (39.53, 45.93)
	Inner regional	5.67 (4.72, 6.63)	42.71% (39.47, 45.97)
	Outer regional and remote	5.57 (4.58, 6.57)	41.58% (38.20, 44.97)
Body Mass Index	Thin	5.90 (4.98, 6.81)	43.31% (40.15, 46.46)
	Healthy weight	6.05 (5.16, 6.94)	42.17% (39.14, 41.26)
	Overweight	6.02 (5.11, 6.92)	42.69% (39.57, 45.81)
	Obese	6.33 (5.39, 7.28)	44.70% (41.46, 47.93)
Achieving Physical Activity guidelines	No	5.40 (4.52, 6.28)	43.52% (40.43, 46.62)
	Yes	6.63 (5.73, 7.52)	41.24% (38.10, 44.38)

Holm’s Sequential Bonferroni adjustment to estimated means and 95% Confidence Intervals (CI) were made

The unadjusted annual mean cost of structured physical activity participation was AUD$1250 p.a. The Active Kids voucher, valued at $100 p.a., supported on average 19.82% [95%CI 17.71, 21.95] of annual expenditure for all study participants. The contribution of the voucher to total expenditure was greater for children who were male (20.54% [95%CI 19.04, 22.05]) compared to female (17.71% [95%CI 16.20, 19.23]); Aboriginal and/or Torres Strait Islander (23.70% [95%CI 21.50, 25.91]) compared to non-Indigenous (18.67% [95%CI 16.60, 20.73]); Children with a disability (21.68% [95%CI 19.76, 23.60]) compared to no disability (19.85% [95%CI 18.15, 21.55]); Children living in the most disadvantaged areas (23.44% [95%CI 21.02, 25.84]) compared to least disadvantaged areas (17.67% [95%CI 15.27, 20.06); Children living in regional (18.79% [95%CI 13.61, 24.00]) or remote areas (22.27% [95%CI 16.92, 27.64]) compared to children living in cities (16.04% [95%CI 10.88, 21.18); and obese children (21.37% [95%CI 19.13, 23.62]) compared to children in the healthy weight range (18.88% [95%CI 16.78, 20.97]).

### Personal and social associations of children with high activity levels

Results comparing children who reported achieving ≥5 days with 60 min of physical activity (*n* = 24,268) with low active children (< 5 days, *n* = 17,394) are displayed in Table 4. More active children had greater odds of their parents correctly recalling children’s physical activity guidelines and their parents being physically active themselves compared with less active children. High active children had supportive home environments and had been active with their parents/carers (especially female parents/carers), siblings, relatives and friends. Active children reported higher self-efficacy than their inactive counterparts, to specifically choose to be active in their free time and reported finding it easy to find and participate in physical activity if they wanted to. Children who were active on ≥5 days per week reported finding physical activity fun; being happy and full of energy significantly more and were less likely to report feelings of loneliness or be unable to concentrate than less active participants.

**Table 4:** High and increased physical activity after voucher use and associated personal/social factors

		Prevalence among all children (***n*** = 32,250)	Binomial comparisons from 32,250 children using 41,662 observations	
			Model 1 - Observations of children active ≥ 5 days per week (***n*** = 24,268), compared to those active on < 5 days per week (***n*** = 17,394) after voucher use	Model 2 - Observations from children who increased the number of days they achieve 60 min of physical activity (***n*** = 12,853), compared to those who maintained same level (***n*** = 24,711) or decreased (***n*** = 4098) days achieving 60 min
		%	Odds Ratio (95%CI)	Odds Ratio (95%CI)
Knows children’s physical activity guidelines	No	51.65	Ref	Ref
	Yes	48.35	1.41 (1.35, 1.48)	0.90 (0.86, 0.95)
Adult achieves physical activity guidelines	No	75.41	Ref	Ref
	Yes	24.59	1.73 (1.65, 1.82)	0.89 (0.84, 0.93)
Adult sport participation	0 sessions	45.06	Ref	Ref
	1 session	14.62	0.88 (0.82, 0.93)	1.01 (0.95, 1.08)
	2 sessions	13.55	0.94 (0.88, 1.00)	0.97 (0.91, 1.04)
	3 sessions	10.86	1.01 (0.94, 1.08)	1.05 (0.98, 1.13)
	4 sessions	6.46	1.08 (0.99, 1.18)	1.07 (0.98, 1.17)
	5 sessions	4.80	1.40 (1.26, 1.55)	1.06 (0.96, 1.18)
	6 sessions	2.02	1.35 (1.16, 1.58)	1.08 (0.93, 1.26)
	7 sessions	1.41	1.55 (1.28, 1.88)	0.88 (0.72, 1.07)
	8+ sessions	1.21	1.53 (1.24, 1.89)	0.78 (0.63, 0.98)
Child’s companions for physical activity at home in the past week*	No companion	11.52	Ref	Ref
	Whole family together	58.54	0.88 (0.84, 0.92)	0.29 (0.27, 0.30)
	Male adult carer	33.52	1.04 (1.00, 1.09)	0.34 (0.32, 0.36)
	Female adult carer	32.65	1.21 (1.15, 1.26)	0.56 (0.54, 0.59)
	Grandparents	22.56	1.02 (0.97, 1.08)	0.18 (0.16, 0.19)
	Siblings	32.37	1.52 (1.45, 1.59)	1.76 (1.68, 1.84)
	Relatives (e.g. cousins)	8.96	1.38 (1.28, 1.49)	1.31 (1.22, 1.42)
	Friends	37.48	1.66 (1.59, 1.74)	2.41 (2.30, 2.52)
Ease/Difficulty of locating places for the child to be physically active	Difficult	9.64	Ref	Ref
	Easy	90.36	1.65 (1.52, 1.79)	0.69 (0.64, 0.75)
The child finds being physically active fun	Disagree	1.88	Ref	Ref
	Neither agree nor disagree	3.51	1.31 (1.09, 1.58)	0.97 (0.80, 1.16)
	Agree	94.61	2.95 (2.53, 3.44)	1.02 (0.88, 1.19)
Self-efficacy to be active during free time	Disagree	4.52	Ref	Ref
	Neither agree nor disagree	9.15	1.30 (1.16, 1.46)	1.16 (1.03, 1.30)
	Agree	86.33	2.83 (2.56, 3.12)	1.05 (0.95, 1.16)
Self-efficacy to ask an adult (parent, carer) to be physically active with them	Disagree	5.01	Ref	Ref
	Neither agree nor disagree	10.93	1.19 (1.07, 1.32)	0.84 (0.75, 0.94)
	Agree	84.00	1.67 (1.53, 1.83)	0.76 (0.69, 0.83)
Self-efficacy to ask a friend to be physically active with them during their free time	Disagree	6.32	Ref	Ref
	Neither agree nor disagree	10.61	1.27 (1.15, 1.40)	1.00 (0.90, 1.10)
	Agree	83.07	1.86 (1.72, 2.02)	0.84 (0.77, 0.91)
Happiness	Unhappy	7.60	Ref	Ref
	Neither happy nor unhappy	4.06	0.70 (0.62, 0.80)	10.98 (9.15, 13.18) **
	Happy	88.35	1.39 (1.29, 1.49)	8.98 (7.69, 10.49) **
Full of energy	Never/Rarely	8.00	Ref	Ref
	Quite often /Always	92.00	3.45 (3.12, 3.82)	1.32 (0.88, 1.97)
Feels lonely	Never/Rarely	92.05	Ref	Ref
	Quite often /Always	7.95	0.49 (0.44, 0.55)	0.81 (0.73, 0.90)
Unable to concentrate	Never/Rarely	83.33	Ref	Ref
	Quite often /Always	16.67	0.64 (0.59, 0.69)	0.87 (0.81, 0.94)

* Participants could select all companions the child had for physical activity, **Due to a low number of children in the comparison group in this model, these values should be interpreted with caution

### Personal and social associations of children who increased active days after voucher use

Children who increased the number of days doing 60 min of physical activity after voucher use (*n* = 12,853) were compared to children who maintained (*n* = 24,711) or decreased (*n* = 4098) the number of days doing 60 min of physical activity from registration (Table 4). Children who increased their active days reported finding physical activity fun, but reported lower self-efficacy to be active in their free time and were more likely to find it difficult to participate in physical activity if they wanted to. Children who increased their physical activity after using and Active Kids voucher reported feeling significantly happier and full of energy and were less likely to report feelings of loneliness or be unable to concentrate, than those who maintained or decreased their activity (Table 4).

## Discussion

Large-scale interventions which reduce barriers to participation in structured physical activity faced by children and adolescents are essential to addressing the global physical inactivity crisis. To overcome cost barriers, implementation of financial incentives for structured physical activities by the public sector appear to be increasing yet, process and outcome evaluations are rarely undertaken [14, 15]. This natural experiment used a prospective cohort study to understand impacts of the state-wide implementation of the universal Active Kids voucher program, a financial incentive intervention, on children’s physical activity participation. At registration for the Active Kids program, less than one in five children met physical activity guidelines. Our results indicate that weekly physical activity increased following the use of an Active Kids voucher and these increases continued over a six-month period. Physical activity guidelines recommend children achieve at least 60 min of moderate to vigorous physical activity seven days per week. The increase from four to five days per week in this population-wide sample demonstrates a significant improvement in physical activity levels through implementation of the Active Kids program. The economic burden of preventable, non-communicable disease associated with physical inactivity is substantial [46, 47]. The implementation of the scaled up state-wide universal Active Kids program shows promise to increase physical activity participation in children and adolescents.

During the first year of implementation, changes in Active Kids participants physical activity levels from registration were positive across all sociodemographic characteristics. In the short term, inequities in physical activity participation fluctuated among sociodemographic groups and after six months, gender inequities had reduced. Female children and adolescents’ physical activity levels increased towards their male counterparts’ levels. It is unclear why females responded substantially better to the Active Kids voucher universal intervention. Though this positive change was observed within sex, disparities remained consistent for other characteristics and grew within language and age characteristic groups. Previous research in Canada using financial incentives for sport found offering the same tax-credit to the whole population disproportionally helped high socioeconomic groups [21]. The Active Kids program has demonstrated high reach and engagement [27]; however socio-economically disadvantaged children, children who speak a primary language other than English at home, obese children and 15–18 year old groups and children in major cities appear to demand additional interventions. Targeted or proportionate universalist approaches that reduce inequities in children and adolescents’ physical activity levels and ensure equitable benefits from Active Kids voucher use require attention [1, 48].

Modifiable barriers to structured physical activity for children and young people are complex and difficult to overcome [9–11]. Cost, access, time and social support affect children and adolescent’s physical activity to different degrees depending on their context. This intervention explicitly targeted cost of structed physical activity participation by providing one Active Kids voucher during the calendar year. Participants reported higher annual expenditure on structured physical activity than population estimates [32], with the Active Kids voucher universally supporting 20% of the structured activity costs. This reflects the underrepresentation of children from low socio-economic areas registered in the Active Kids program [26]. The contribution of the Active Kids voucher to annual expenditure achieved was similar to previous estimates [15]. Significantly greater contributions were observed among children living in disadvantaged areas compared to advantaged areas, however the dose-response relationship estimated by Reece et al. using population medians was far smaller in our natural experiment [15]. The Active Kids voucher supported two fifths of children’s weekly time participating in structured physical activities. This is the first study to report the contribution of voucher-specific activity to children and adolescent’s total time participating in structured physical activity. The contribution of the voucher to expense and structured physical activity duration suggests that children and adolescents who used an Active Kids voucher are participating in a variety of structured physical activities rather than specialising in one, which is ideal for ongoing participation [49]. Vella et al. has previously highlighted that structured physical activity participation alone is not enough to accrue health benefits of physical activity [50]. Our data also shows that children with lower self-efficacy to be active in their free time and those who found it difficult to participate in physical activity increased their physical activity levels after using a voucher. The Active Kids voucher makes a clear contribution to participation in structured physical activity for children and adolescents in NSW, reducing (but not removing) the cost barrier to structured physical activity participation and with reduced cost barriers, also increasing their physical activity levels.

There is strong evidence that comprehensive, multi-component strategies are required to increase physical activity and prevent non-communicable disease [1, 48]. The ACTIVE trial included peer mentoring and support worker engagement components in addition to the financial incentives, although these were unsuccessful [18]; James et al. reported a need to overcome accessibility barriers [18]. Scalable components which address modifiable barriers, in addition to cost, such as mass-media campaigns and enhanced active travel infrastructure have not been investigated with financial incentive interventions to date.

Regular participation in structured physical activity outside of school has immediate and long-term benefits for children’s development, educational attainment, physical, psychological and social health [2, 4, 51]. Participation during childhood is predictive of a lasting commitment to engage in structured physical activity [7, 8]. Previous research has shown that children and adolescents whose caregivers know children’s physical activity guidelines and achieve the physical activity guidelines for adults themselves, are more likely to be Active Kids [52, 53]. This was also true in our study population with female adult caregivers having a stronger association with high activity than male adult carers, grandparents or the whole family together. Children who achieved ≥5 days of moderate-to-vigorous physical activity per week were more likely have self-efficacy to be active alone and with others, more likely to be happy, be able to concentrate, and less likely to express feelings of loneliness. Children who were highly active and who increased their active days during their participation in the Active Kids program more likely to be physically active with other children (siblings/friends/teammates/cousins). Social connections developed through hours of structured physical activity participation during the Active Kids program could be associated with higher physical activity levels. These findings demonstrate the strength of social support for initiating and sustaining physical activity, and how essential interpersonal relationships between young people are in positively influencing physical activity participation. Fostering the development of social connections during structured physical activity may provide additional health enhancing benefits.

Program design features of financial incentive interventions for youth physical activity participation have been varied in all settings of implementation [18–21]. Features such as the target population, administration process, activity eligibility, activity duration, and amount of financial support are likely to moderate the effectiveness of these incentives. The Active Kids program targeted all school-enrolled children and, in Australia, was innovative as it broadened from sport to include all structured physical activities, as in the Canadian Fitness Tax-Credit [21]. Prior to this, interventions by the NSW government, Australia with structured physical activity providers were mostly with sports organisations. The Active Kids program was the only known financial incentive program internationally to accredit eligible activities, to ensure they provided moderate-to-vigorous physical activity and lasted for at least 8 weeks. This allowed a diverse range of providers from across the state to register as an activity provider in the program, rather than known sports organisations, perhaps appealing to children who may not be interested in sport but were considering other structured physical activities. Notably though, the Active Kids vouchers could not be used for school holiday programs (duration < 8 weeks) or programs held during school time or delivered by schools [29]. The definition of structured physical activities, the duration of program, and point of sale financial support provided by the Active Kids voucher were central to the high community reach [26], and improvements in physical activity behaviours. The ACTIVE trial has highlighted the importance of ensuring incentivised activities align with adolescents personal preferences [18]. Although this is a more resource intensive approach, it is a promising strategy for populations who are hard to reach.

Collectively, this population-wide study has implications for public policy maker efforts to increase physical activity participation in children and young people. The prospective cohort study design which explored outcomes in using natural experimentation suggest that these results are may be generalisable to similar populations. The Active Kids program includes a substantial sample of NSW children, compared to Census data, which allows us to provide confident estimates of the outcomes achieved through the program [26]. Although the study sample was generally representative of all children who used an Active Kids voucher (Table 1), limitations exist. Consistent with other natural experiments of policy interventions and the scale of the Active Kids program, we were unable to establish a comparison group [28]. The cohort participants reflect a bias towards a healthier more active population, especially those who completed two surveys, with underrepresentation from children living in socio-economically disadvantaged areas, obese children and children who casually participated in sport in the 12 months before registration. Older adolescents were under-represented in the sample and of those participating, adolescents who used an Active Kids voucher were more active and engaged in sport at registration. The online questionnaire was the most pragmatic measurement tool, however we acknowledge that the use of self-report data (often reported by-proxy through parent/guardian) is prone to social desirability bias and recall bias [39]. There is potential that pre-test sensitization may have inflated the effects of the intervention through repeated use of the measurement tool, however this cannot be estimated. Future studies should strive to use device-based measurement to monitor change in physical activity. Finally, data were collected using validated self-report or proxy-report items where possible; items for all ages (4.5–18 years) included in our study however if adults were completing the survey without the child present, social and wellbeing items were skipped to strengthen internal validity. Further research should continue to strengthen the tools available for the evaluation of scaled-up interventions for children of all ages. The pragmatic approach in the evaluation of this natural experiment was central to beginning to understand the long-term influences of the Active Kids voucher program children and adolescents.

## Conclusion

The Active Kids program reduced the cost of structured physical activity for children and adolescents in NSW and significantly increased children’s physical activity levels up to at least 6 months after voucher use. Thereby, the Active Kids program shows promise as a scaled-up effective intervention to increase children and adolescents’ physical activity participation. This study provides unique and policy-informing insight into how state-wide public sector financial incentives can positively effect children and adolescents’ physical activity behaviours, and the associated economic, personal and social impacts. Further work is needed across government and in the private sector to leverage Active Kids to successfully reduce inequities in children and adolescents’ physical activity levels and increase the proportion of school-aged children achieving health enhancing physical activity levels.

## Supplementary Information

**Additional file 1:.** Active Kids evaluation survey question items

**Additional file 2:.** Multivariable analysis for number of days doing 60 min physical activity

## Data Availability

The datasets used and/or analysed during the current study are available from the corresponding author on reasonable request.

## Abbreviations

NSW: New South Wales
BMI: Body Mass Index z-scores
95%CI: 95% Confidence Interval
