# Supplementary figures and images for: Effects of the Active Kids voucher program on children and adolescents’ physical activity: a natural experiment evaluating a state-wide intervention
Source: BMC Public Health. 2021 Jan 11;21:22. doi: 10.1186/s12889-020-10060-5 (PMC7798231; doi:10.1186/s12889-020-10060-5)

# Additional File 1. Active Kids evaluation survey question items


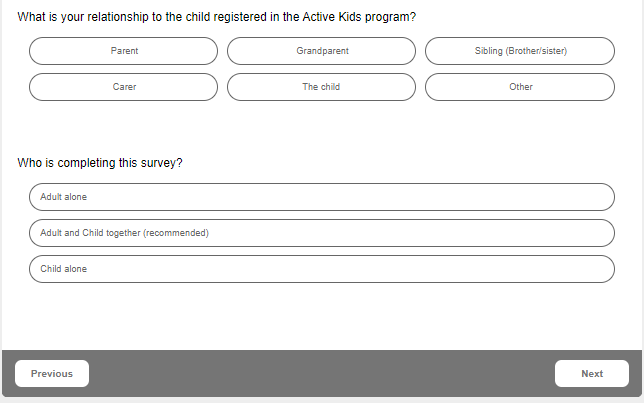


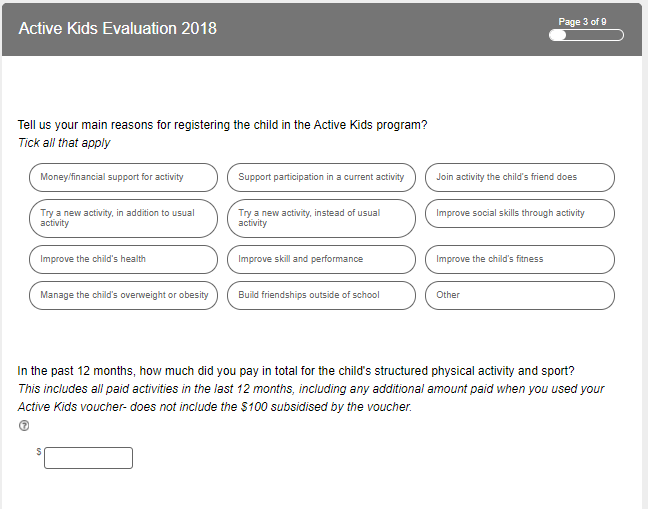


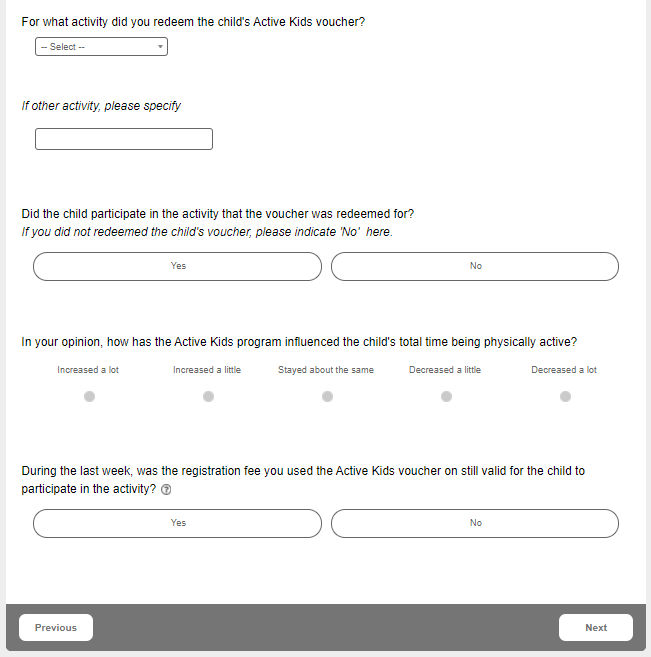


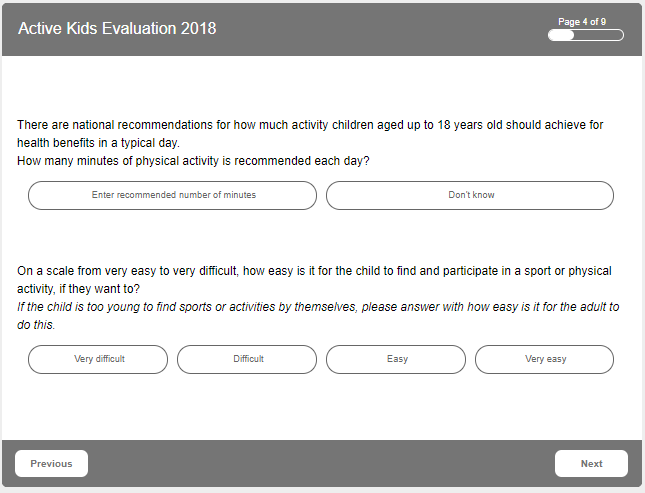


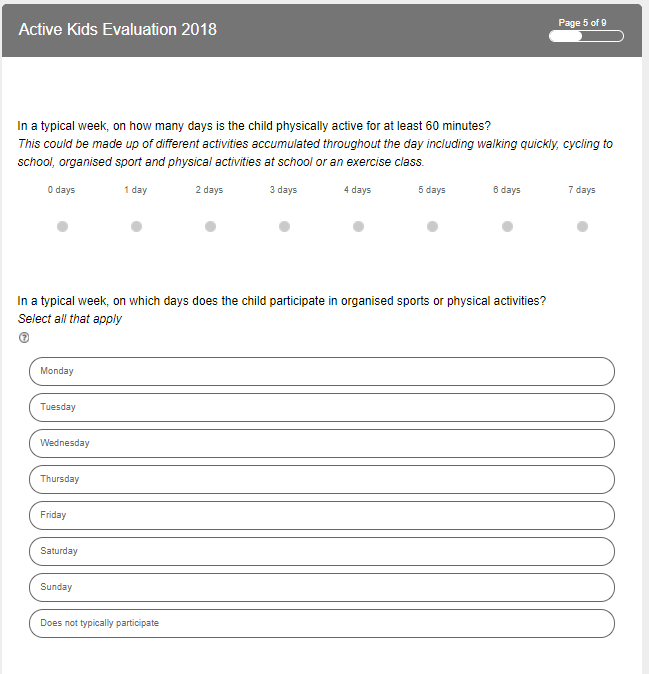


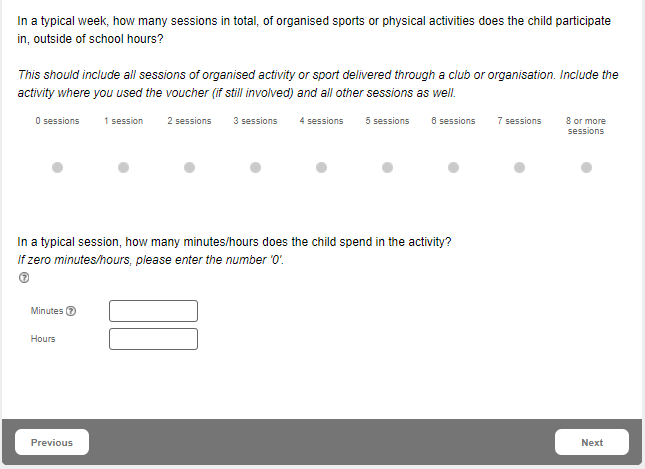


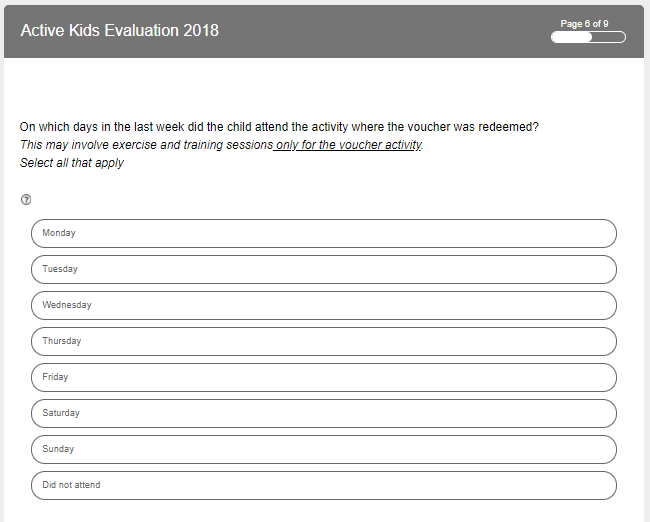


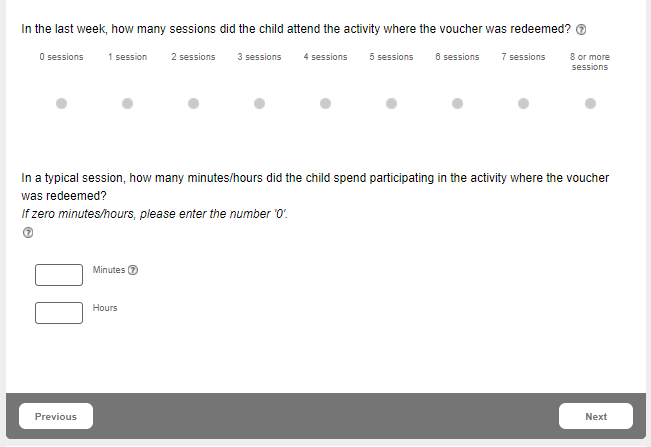


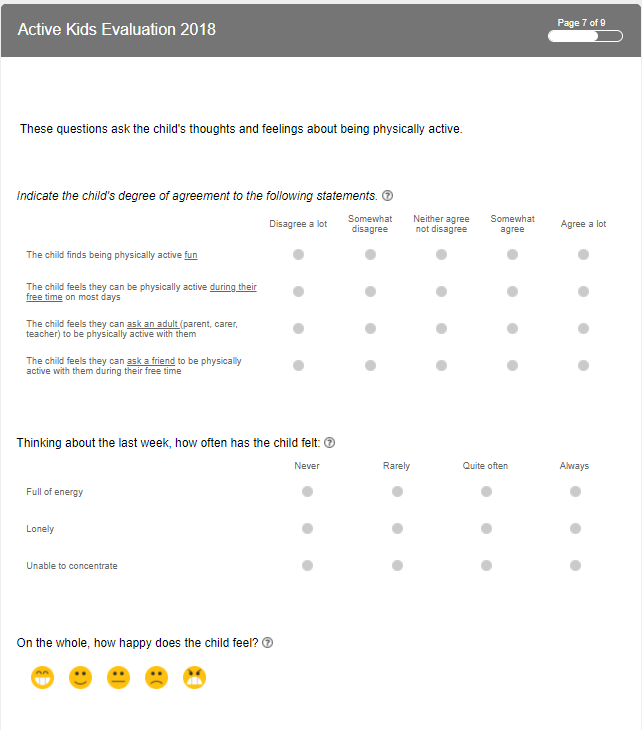


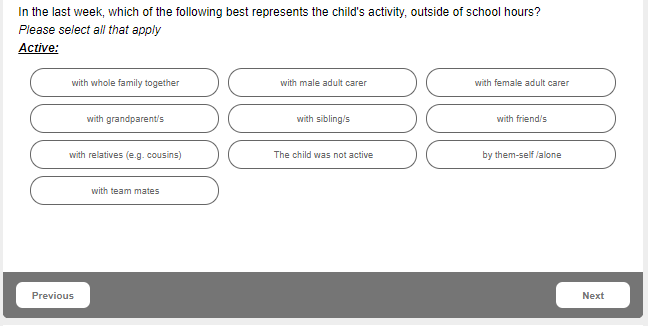


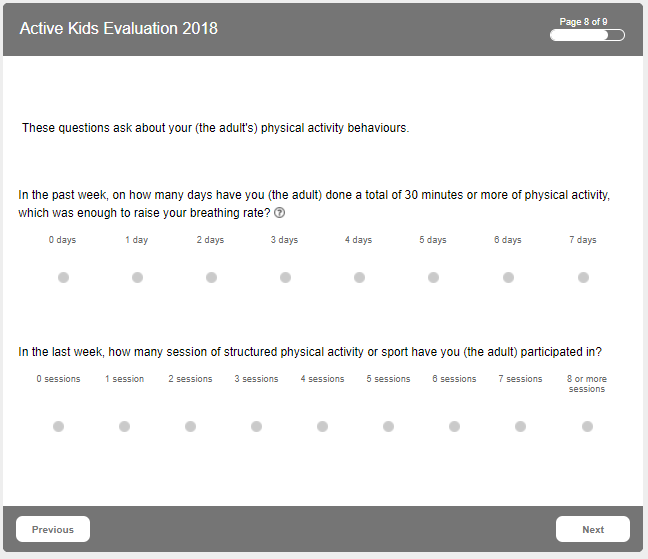


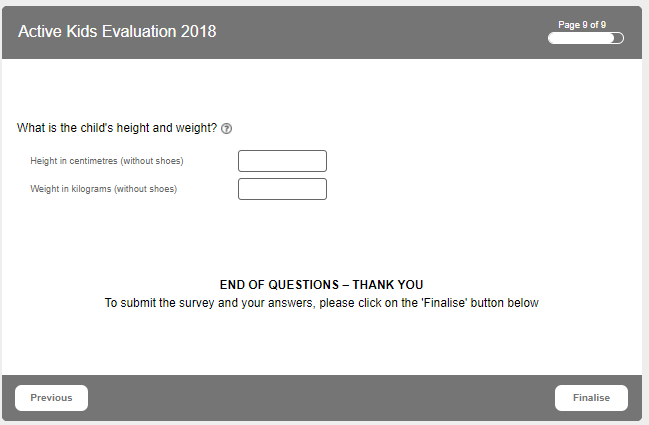

Supplement: Supplementary file 1 — Additional file 1:. Active Kids evaluation survey question items [file 12889_2020_10060_MOESM1_ESM.docx]
