# Supplementary material for: Effects of the Active Kids voucher program on children and adolescents’ physical activity: a natural experiment evaluating a state-wide intervention
Source: BMC Public Health. 2021 Jan 11;21:22. doi: 10.1186/s12889-020-10060-5 (PMC7798231; doi:10.1186/s12889-020-10060-5)
Supplement: Supplementary file 2 — Additional file 2:. Multivariable analysis for number of days doing 60 min physical activity [file 12889_2020_10060_MOESM2_ESM.docx]

**Additional File 2 -Multivariable analysis for number of days doing 60 minutes physical activity**

|  |  | Coefficient | Lower 95% Confidence Interval | Upper 95% Confidence Interval | P value |
| --- | --- | --- | --- | --- | --- |
| Timepoint | Registration | Ref |  |  |  |
|  | 8 weeks or less | 0.247 | 0.219 | 0.273 | <0.0001 |
|  | 9-26 weeks | 0.299 | 0.282 | 0.317 | <0.0001 |
|  | 27 weeks + (6 months+) | 0.934 | 0.909 | 0.957 | <0.0001 |
| Sex | Male | Ref |  |  |  |
|  | Female | -0.324 | -0.355 | -0.290 | <0.0001 |
| Age group | 4-8 years | Ref |  |  |  |
|  | 9-11 years | -0.058 | -0.098 | -0.019 | 0.004 |
|  | 12-14 years | -0.217 | -0.261 | -0.171 | <0.0001 |
|  | 15-18 years | -0.367 | -0.421 | -0.311 | <0.0001 |
| Aboriginal/Torres Strait Islander | No | Ref |  |  |  |
|  | Yes | 0.058 | -0.024 | 0.140 | 0.166 |
| Primary language spoken at home | English | Ref |  |  |  |
|  | Language other than English | -0.631 | -0.692 | -0.570 | <0.0001 |
| Identified disability | No | Ref |  |  |  |
|  | Yes | -0.486 | -0.582 | -0.388 | <0.0001 |
| Socio-economic status * | 1st Quartile (Most Disadvantaged) | Ref |  |  |  |
|  | 2nd Quartile | 0.205 | 0.147 | 0.264 | <0.0001 |
|  | 3rd Quartile | 0.189 | 0.131 | 0.247 | <0.0001 |
|  | 4th Quartile (Least Disadvantaged) | 0.320 | 0.262 | 0.378 | <0.0001 |
| Geographic location | Major city | Ref |  |  |  |
|  | Inner regional | 0.213 | 0.168 | 0.259 | <0.0001 |
|  | Outer regional and remote | 0.337 | 0.255 | 0.421 | <0.0001 |
| Body Mass Index | Thin | -0.012 | -0.079 | 0.054 | 0.728 |
|  | Healthy weight | Ref |  |  |  |
|  | Overweight | -0.234 | -0.292 | -0.175 | <0.0001 |
|  | Obese | -0.537 | -0.624 | -0.449 | <0.0001 |
